# A new species of the genus *Deinodryinus* Perkins (Hymenoptera, Dryinidae) from the USA

**DOI:** 10.3897/zookeys.809.30647

**Published:** 2018-12-19

**Authors:** Stefano Speranza, Massimo Olmi, Adalgisa Guglielmino, Mario Contarini

**Affiliations:** 1 Department of Agriculture and Forest Sciences (DAFNE), University of Tuscia, Viterbo, Italy University of Tuscia Viterbo Italy; 2 Tropical Entomology Research Center, Viterbo, Italy Tropical Entomology Research Center Viterbo Italy

**Keywords:** Chrysidoidea, Anteoninae, Texas, Buescher State Park, *
Deinodryinus bimaculatus
*, *
Deinodryinus masneri
*, taxonomy, key

## Abstract

A new species of *Deinodryinus* Perkins, 1907, is described from the USA, Texas: *D.bimaculatus***sp. n**. Morphologically the new species is similar to *D.masneri* (Olmi, 1984), but it is distinguished by the head lacking a frontal line and the forewing crossed by two dark transverse bands; in *D.masneri* the head shows a conspicuous frontal line and the forewing is hyaline and without dark transverse bands.

## Introduction

Dryinidae (Hymenoptera: Chrysidoidea) are parasitoids and often also predators of leafhoppers, planthoppers and treehoppers (Hemiptera, Auchenorrhyncha) (Guglielmino et al. 2013). They comprise 16 subfamilies, 50 genera and more than 1800 world species (Olmi and Xu 2015; Tribull 2015).

One of the most common genera of this family is *Deinodryinus* Perkins, 1907, belonging to the subfamily Anteoninae. *Deinodryinus* species are parasitoids of leafhoppers belonging to the Cicadellidae (Guglielmino et al. 2013; Olmi and Virla 2014; Olmi and Xu 2015). As in almost all dryinids, females of *Deinodryinus* have a chelate protarsus. Chelae are used to capture and restrain the host during oviposition and host feeding (Olmi 1984, 1994).

According to Olmi (1984, 1987), in the Nearctic region, the genus *Deinodryinus* includes four species. In 2017 the authors examined a further new species collected in Texas. It is described below.

## Material and methods

The description follows the terminology used by Olmi (1984), Guglielmino et al. (2017b, 2018) and Olmi and Virla (2014). The measurements reported are relative, except for the total length (head to abdominal tip, without the antennae), which is expressed in millimeters. In the descriptions, POL is the distance between the inner edges of the lateral ocelli; OL is the distance between the inner edges of a lateral ocellus and the median ocellus; OOL is the distance from the outer edge of a lateral ocellus to the compound eye; OPL is the distance from the posterior edge of a lateral ocellus to the occipital carina; TL is the distance from the posterior edge of an eye to the occipital carina.

The term “metapectal-propodeal complex” is here used in the sense of Kawada et al. (2015). It corresponds to the term “metathorax + propodeum” sensu Olmi (1984), Xu et al. (2013), Olmi and Virla (2014) and Olmi and Xu (2015). The terms “metapostnotum” and “first abdominal tergum” sensu Kawada et al. (2015), used here, correspond to the terms “dorsal surface of propodeum” and “posterior surface of propodeum”, *sensu*Olmi (1984), Xu et al. (2013), Olmi and Virla (2014) and Olmi and Xu (2015).

The types of all Nearctic species of *Deinodryinus* were examined. The material studied in this paper is deposited in the collection of the Department of Entomology, Texas A&M University, College Station, Texas, USA (TAMU).

The description of the new species is based on the study of only a single specimen. The authors are aware that descriptions of new taxa should normally be based on more individuals. However, Dryinidae are so rare that it is uncommon to collect more than one specimen of each species. In addition, on the basis of the experience and knowledge of the authors, the new species is sufficiently delimited by unique characters to justify its description.

## Results

### Genus Deinodryinus

Taxon classificationAnimaliaHymenopteraDryinidae

Perkins, 1907

Deinodryinus Perkins, 1907: 45.

#### Type species.

*Deinodryinusparadoxus* Perkins, 1907, designated by Muesebeck and Walkley (1951).

#### Diagnosis.

**Figure 1.**
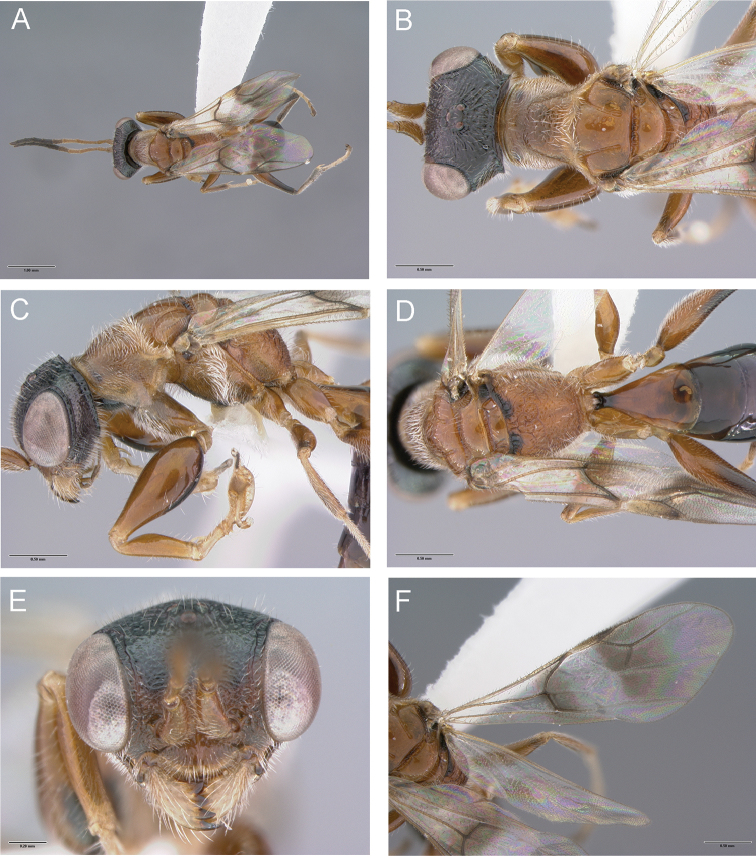
*Deinodryinusatriventris* (Cresson), female from Ohio, Columbus: **A** habitus in dorsal view **B** head and mesosoma in dorsal view **C** head and mesosoma in lateral view **D** metapectal-propodeal complex in dorsal view **E** head in frontal view **F** forewing.

Female (Fig. 1): Macropterous or micropterous; palpal formula 6/3; occipital carina complete; vertex of head frequently with two strong oblique keels connecting posterior ocelli to occipital carina; pronotum with distinct anterior collar and posterior disc; in macropterous females forewing usually with distal part of stigmal vein longer than proximal part, less frequently as long as, or shorter than proximal part; enlarged claw with inner proximal prominence not bearing bristles, with one or two bristles or peg-like hairs located further distally than proximal prominence; tibial spurs 1/1/2. Male: Macropterous (even with micropterous female); palpal formula 6/3; vertex of head frequently with two strong oblique keels connecting posterior ocelli to occipital carina; antennal hairs usually much longer than breadth of segments, less frequently shorter than breadth of segments; forewing usually with distal part of stigmal vein longer than proximal part, less frequently as long as, or shorter than proximal part; forewing usually with pterostigma four, or more than four, times as long as broad; paramere without dorsal process, usually with one more-or-less large inner branch wrapping penis, less frequently with one reduced inner branch; tibial spurs 1/1/2.

### Deinodryinus bimaculatus sp. n.

Taxon classificationAnimaliaHymenopteraDryinidae

http://zoobank.org/229725A4-497A-40D7-AE11-3ED7BCE6D7AB

Fig. 2

#### Diagnosis.

**Figure 2.**
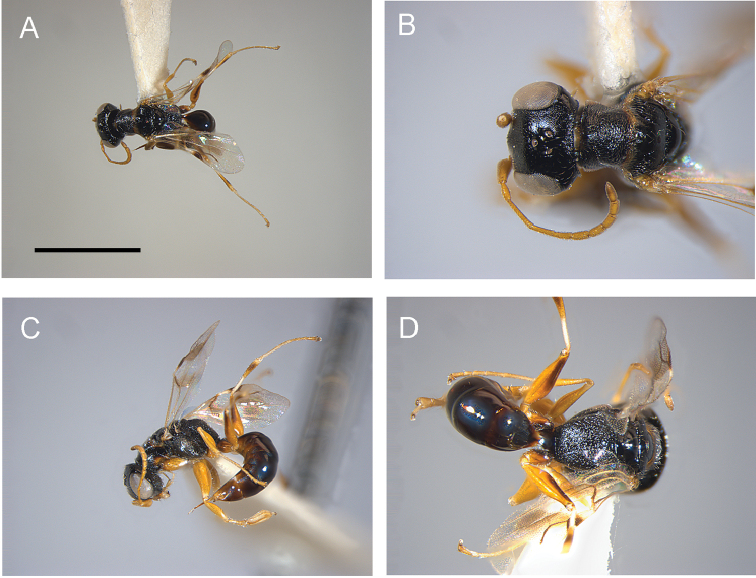
*Deinodryinusbimaculatus* sp. n., female holotype: **A** habitus in dorsal view **B** head, pronotum and mesoscutum in dorsal view **C** habitus in lateral view **D** metapectal-propodeal complex in dorsal view. Scale bars: 2.09 mm (**A**), 0.95 mm (**B**), 1.50 mm (**C**), 0.99 mm (**D**).

Female with head not provided with two oblique keels connecting posterior ocelli to occipital carina (Fig. 2B); head without frontal line (Fig. 2B); forewing with two dark transverse bands (Fig. 2C); metapectal-propodeal complex strongly reticulate rugose, mainly on metapostnotum (Fig. 2D).

#### Description.

**Figure 3.**
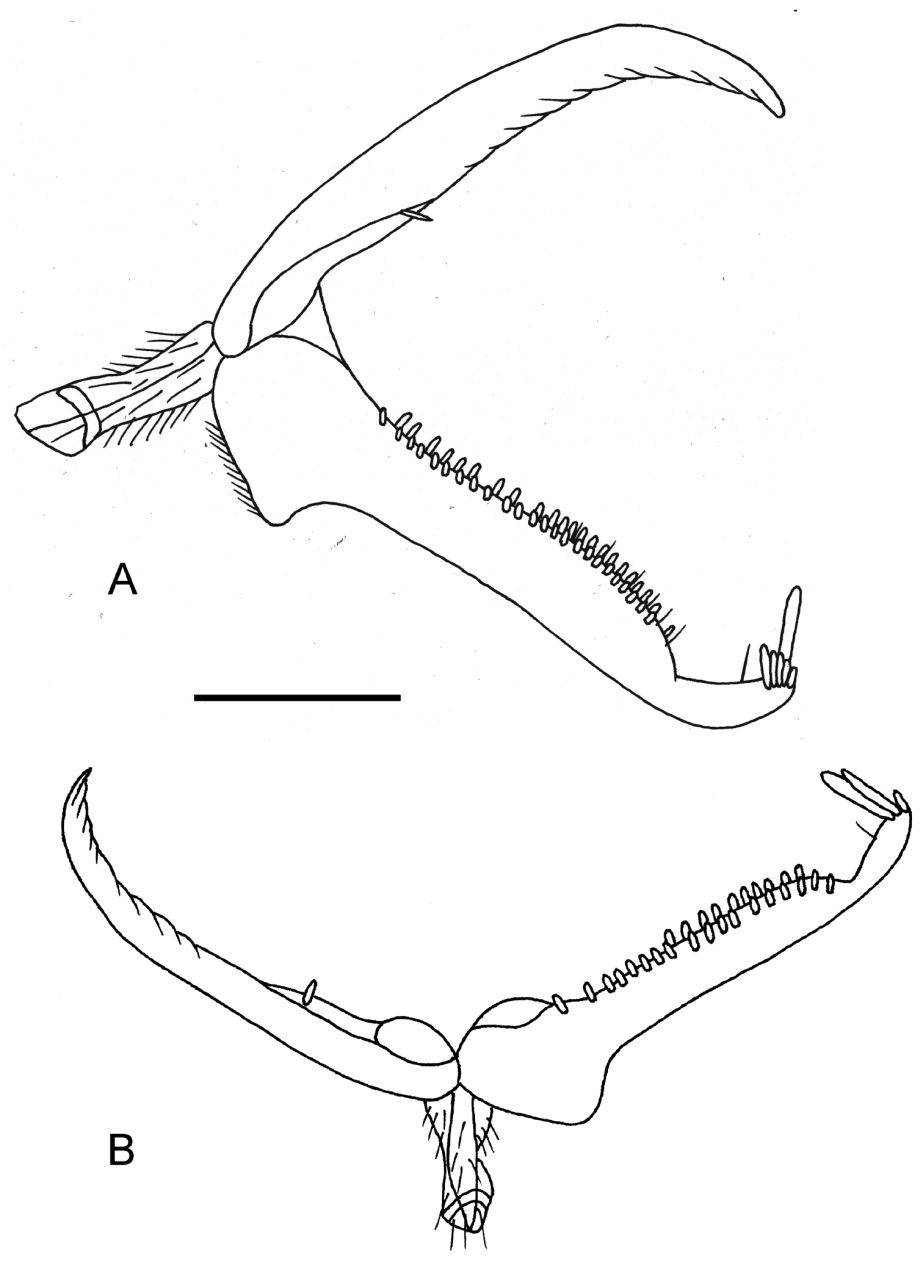
*Deinodryinusbimaculatus* sp. n., female holotype: **A** chela **B***Deinodryinusmasneri* (Olmi), female from California, Tulare Co., Clough’s Cave; chela. Scale bars: 0.08 mm (**A**), 0.12 mm (**B**).

**Female.** Fully winged (Fig. 2). Length 2.4 mm. Head black, except mandible, clypeus and gena testaceous. Antenna testaceous, except antennomere 10 slightly darkened. Mesosoma black, except anterior, posterior and lateral margins of pronotum testaceous. Metasoma brown. Legs testaceous, except metacoxa partly brown, club of metafemur and metatibia with brown spot. Antenna clavate. Antennomeres in following proportions: 9:5:10:7:5:5:5:5:5:6. Head dull, granulate (Fig. 2B). Frontal line absent. Occipital carina complete. Head without oblique keels connecting posterior ocelli to occipital carina (Fig. 2B). POL = 3; OL = 3; OOL = 5; OPL = 6; TL = 6. Greatest breadth of lateral ocelli shorter than OPL (2:6). Pronotum shiny, punctate, unsculptured among punctures, sculptured by many transverse striae, with posterior surface about as long as mesoscutum. Mesoscutum shiny, very slightly granulate, mainly on lateral regions. Notauli incomplete (Fig. 2B), reaching approximately 0.6 × length of mesoscutum. Mesoscutellum and metanotum shiny, unsculptured. Metapectal-propodeal complex dull, reticulate rugose and granulate, without transverse or longitudinal keels (Fig. 2D). Metapostnotum and first abdominal tergum not separated by transverse keel. Forewing hyaline, with two dark transverse bands (Fig. 2D). Distal part of stigmal vein longer than proximal part (8:6). Protarsomeres in following proportions: 7:3:4:10:18. Enlarged claw (Fig. 3) with one bristle situated further distally than proximal prominence. Protarsomere 5 (Fig. 3) with two rows of about 46 lamellae and distal apex provided with approximately six lamellae, among which one much longer than others. Tibial spurs 1/1/2.

**Male.** Unknown.

#### Material examined.

**Holotype**: female, USA: Texas, Bastrop Co., Buescher State Park, 29.iv–10.v.1990, R. Wharton leg. (TAMU).

#### Hosts.

Unknown.

#### Distribution.

USA (Texas).

#### Etymology.

The species is named *bimaculatus* (adjective formed by the prefix “bi-“ meaning “two” + the Latin adjective “maculatus”, meaning “spotted”), because its forewing shows two dark transverse bands.

#### Remarks.

On the basis of the morphological characters indicated in the above diagnosis, *D.bimaculatus* is similar to *D.masneri* (Olmi, 1984), but it differs because the head has no frontal line (frontal line present in *D.masneri*) and the forewing has two dark transverse bands (no dark bands in *D.masneri*). Following the description of the new species, the key to the females of Nearctic *Deinodryinus* published by Olmi (1984) can be revised as follows:

**Table:** 

1	Metapectal-propodeal complex strongly reticulate rugose, mainly on metapostnotum (Figs 1D, 2D)	**2**
–	Metapectal-propodeal complex not reticulate rugose; metapostnotum surface mainly smooth, except some slight irregular keels (Fig. 4A)	**4**
2	Vertex of head with two complete keels connecting posterior ocelli to occipital carina (Fig. 4B); occasionally keels incomplete (Fig. 1B)	***D.atriventris* (Cresson)**
–	Vertex of head without two oblique keels connecting posterior ocelli to occipital carina (Fig. 2B)	**3**
3	Head with frontal line; forewing hyaline, without dark transverse bands	***D.masneri* (Olmi)**
–	Head without frontal line (Fig. 2B); forewing with two dark transverse bands (Fig. 2C)	***D.bimaculatus* sp. n**.
4	Face mostly smooth, except few irregular keels near clypeus	***D.quercicolus* Perkins**
–	Face completely sculptured by irregular keels (Fig. 4C)	***D.paradoxus* Perkins**

## Conclusion

Olmi (1984, 1987) listed in the Nearctic region the following four species of *Deinodryinus*: *D.atriventris* (Cresson, 1872), known from Canada, Mexico and USA; *D.masneri* (Olmi, 1984), *D.paradoxus* Perkins, 1907, and *D.quercicolus* Perkins, 1907, known from Mexico and USA. Following the above description, *D.bimaculatus* sp. n., from Texas, is added to the previous lists.

The genus *Deinodryinus* comprises now 163 species (including the new species hereby described), recorded in all the zoogeographic regions, except Antarctica. In the Neotropical region, 112 species are known (Olmi and Virla 2014). In the other regions the number of known species is fewer: nine species in the Oriental region (Xu et al. 2013), seven species in the Palaearctic region (unpublished data), 28 species in the Afrotropical region (unpublished data), two species in the Australian region (Olmi 1991); and five species in the Nearctic region (according to the present paper).

The most common Nearctic species of *Deinodryinus* is *D.atriventris*. One of the authors (MO) reared this species in 2002 in New York state (surroundings of Geneva, Ontario Co.) from *Gyponanacacumina* DeLong and *Gyponanalamina* DeLong (Cicadellidae, Gyponinae), well known vectors of phytoplasmas (Hill and Sinclair 2000) (**New host record**; no other hosts are known). The hosts of other Nearctic species of *Deinodryinus* are unknown (Guglielmino et al. 2013). Records of hosts in the genus *Deinodryinus* are very rare. Previously, the unique records were those of Guglielmino et al. (2013, 2017a). They quoted the following hosts: in Italy, *Laburrusquadratus* (Forel) (Cicadellidae, Deltocephalinae) as host of *Deinodryinushispanicus* (Olmi, 1991); in South Africa, *Colistraparvulus* (Linnavuori) (Cicadellidae, Deltocephalinae) as host of *Deinodryinusdanielssoni* Olmi, 1998; in Namibia, *Aconurellacompta* (Naudé), *Exitianusnanus* (Distant) and *Exitianusokahandia* Ross (Cicadellidae, Deltocephalinae) as hosts of *Deinodryinuspaulyi* (Olmi, 1987).

**Figure 4.**
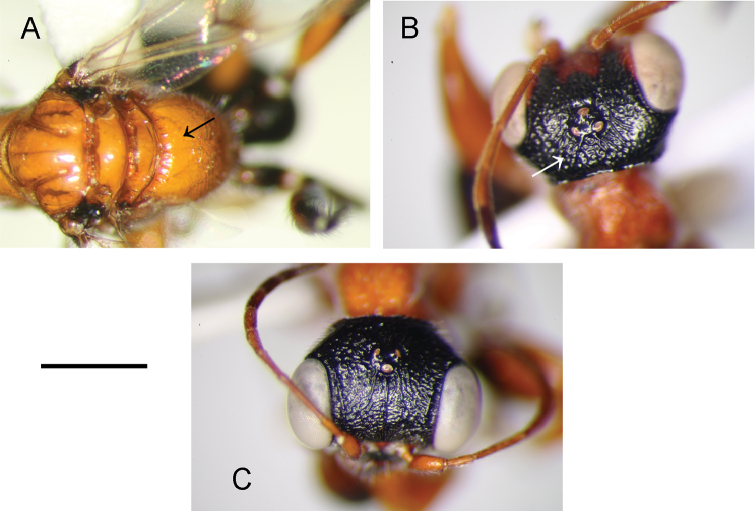
*Deinodryinusparadoxus* Perkins: female from Arizona, Madera Canyon: **A** mesosoma in dorsal view **C** head in dorsal view. *Deinodryinusatriventris* (Cresson), female from Kentucky, Herndon Farm: **B** head in dorsal view. Scale bars: 0.84 mm (**A**), 0.61 mm (**B**), 0.59 mm (**C**).

## Supplementary Material

XML Treatment for Deinodryinus

XML Treatment for Deinodryinus bimaculatus

## References

[B1] Guglielmino A Olmi M Bückle C (2013) An updated host-parasite catalogue of world Dryinidae (Hymenoptera: Chrysidoidea). Zootaxa 3740: 1–113. 10.11646/zootaxa.3740.1.1 25112881

[B2] Guglielmino A Olmi M Marletta A Bückle C (2017a) Larval morphology of three species of Anteoninae (Hymenoptera, Dryinidae). Zootaxa 4320: 470–486. 10.11646/zootaxa.4320.3.4

[B3] Guglielmino A Olmi M Marletta A Speranza S (2017b) Description of the first species of *Gonadryinus* Olmi (Hymenoptera, Dryinidae) from the Afrotropical region. Zootaxa 4238: 440–444. 10.11646/zootaxa.4238.3.11 28603268

[B4] Guglielmino A Olmi M Marletta A Speranza S (2018) Discovery of the first species of *Dryinus* Latreille (Hymenoptera: Dryinidae) from Burmese amber. Zootaxa 4394: 443–448. 10.11646/zootaxa.4394.3.10 29690365

[B5] Hill GT Sinclair WA (2000) Taxa of Leafhoppers Carrying Phytoplasmas at Sites of Ash Yellows Occurrence in New York State. Plant Disease 84(2): 134–138. 10.1094/PDIS.2000.84.2.134 30841302

[B6] Kawada R Lanes GO Azevedo CO (2015) Evolution of metapostnotum in flat wasps (Hymenoptera, Bethylidae): implications for homology assessments in Chrysidoidea PLoS ONE 10(10): e0140051. 10.1371/journal.pone.0140051 PMC4605639 26465151

[B7] Muesebeck CFW Walkley LM (1951) Family Dryinidae. In: Muesebeck CFW Krombein KV Townes HK (Eds) Hymenoptera of America North of Mexico.Synoptic catalogue. U.S. Department of Agriculture, Agriculture Monograph, 2, Washington, D.C., 1034–1043.

[B8] Olmi M (1984) A revision of the Dryinidae (Hymenoptera). Memoirs of the American Entomological Institute 37: i–xii + 1–1913.

[B9] Olmi M (1987) New species of Dryinidae (Hymenoptera, Chrysidoidea). Fragmenta Entomologica 19: 371–456.

[B10] Olmi M (1991) Supplement to the revision of the world Dryinidae (HymenopteraChrysidoidea). Frustula entomologica (NS) 12 [1989]: 109–395.

[B11] Olmi M (1994) The Dryinidae and Embolemidae (Hymenoptera: Chrysidoidea) of Fennoscandia and Denmark (Fauna Entomologica Scandinavica 30). E.J. Brill, Leiden, Netherlands, 100 pp.

[B12] Olmi M Virla EG (2014) Dryinidae of the Neotropical Region (Hymenoptera: Chrysidoidea). Zootaxa 3792: 1–534. 10.11646/zootaxa.3792.1.1 24869997

[B13] Olmi M Xu Z (2015) Dryinidae of the Eastern Palaearctic region (Hymenoptera: Chrysidoidea). Zootaxa 3996: 1–253. 10.11646/zootaxa.3996.1.1 26250320

[B14] Perkins RCL (1907) Parasites of leaf-hoppers. Report of Work of the Experiment Station of the Hawaiian Sugar Planters’ Association, Division of Entomology, Bulletin No. 4: 5–59.

[B15] Tribull CM (2015) Phylogenetic relationships among the subfamilies of Dryinidae (Hymenoptera, Chrysidoidea) as reconstructed by molecular sequencing. Journal of Hymenoptera Research 45: 15–29. 10.3897/JHR.45.5010

[B16] Xu Z Olmi M He J (2013) Dryinidae of the Oriental region (Hymenoptera: Chrysidoidea). Zootaxa 3614: 1–460. 10.11646/zootaxa.3614.1.1 24759692

